# Rising Trends of Endoscopic Barrett’s Esophagus and Gastric Fundic Gland Polyps in Young Japanese Adults

**DOI:** 10.5152/tjg.2023.22533

**Published:** 2023-09-01

**Authors:** Atushi Morita, Akira Horiuchi, Hiroyoshi Ota, Ichitaro Horiuchi, Hidetosi Takada

**Affiliations:** 1 Department of Pediatrics, University of Tsukuba Hospital, Tsukuba, Japan; 2 Digestive Disease Center, Showa Inan General Hospital, Komagane, Japan; 3 Department of Biomedical Laboratory Medicine, Shinshu University Faculty of Medicine, Matsumoto, Japan; 4 Department of Gastroenterology, Shinshu University Faculty of Medicine, Matsumoto, Japan; 5 Department of Pediatrics, University of Tsukuba Hospital, Tsukuba, Japan

**Keywords:** Reflux esophagitis, endoscopic Barrett’s esophagus, gastric fundic gland polyp

## Abstract

**Background/Aims:**

This study examined changes in the esophageal–gastric junction and gastric mucosa in young Japanese adults undergoing endoscopy in the last 15 years.

**Materials and Methods:**

This was a retrospective study of young Japanese adults (aged 19-30 years) who underwent esophagogastroduodenoscopy between 2006 and 2020. The indications were upper gastrointestinal symptoms and anemia. Changes in the appearance of the esophago–gastric junction (i.e., the Z line and distal esophagitis) and gastric mucosa were examined. Endoscopic Barrett’s esophagus was defined using the Japanese criteria.

**Results:**

One thousand eight hundred forty-five patients were examined: 848 from 2006 to 2012 [400 males, mean age 26.5 years (range 19-30)] and 997 from 2013 to 2020 [433 males, mean age 26.2 years (range 19-30)]. The proportion showing endoscopic Barrett’s esophagus and gastric fundic gland polyps increased significantly between the 2 periods (12.5% vs. 22.4%, *P* < .001; 3.4% vs. 7.2%, *P* < .001) with a significant correlation between the prevalence trends for endoscopic Barrett’s esophagus and gastric fundic gland polyps (*r* = 0.789, *P* = .0008). Pathological examination showed that the prevalence of traditional fundic gland polyps unrelated to the use of proton pump inhibitors significantly increased from 40% (4/10) to 81% (25/31) between the 2 periods (*P* = .04).

**Conclusion:**

The prevalence of both endoscopic Barrett’s esophagus and gastric fundic gland polyps among young Japanese adults significantly increased in the last 15 years. The trend in endoscopic Barrett’s esophagus was significantly correlated with that of non-proton pump inhibitor-related gastric fundic gland polyps.

**Main Points**

- The prevalence of both endoscopic Barrett’s esophagus and gastric fundic gland polyps among young Japanese adults has significantly increased in the last 15 years.
- The trend in endoscopic Barrett’s esophagus was significantly correlated with that of gastric fundic gland polyps, which was not linked to the use of proton pump inhibitors.
- This correlation may suggest that higher protein consumption in young Japanese adults increased gastric acid secretion.

## Introduction

The rapid decline in the prevalence of *Helicobacter pylori* infection in young Japanese adults and changes in the diet to one high in protein have resulted in a major change in the type and prevalence of diseases occurring in the proximal gastrointestinal tract. These changes are suggested by the increase in body mass and height of young Japanese, the rapid decline in the prevalence of peptic ulcers, and the increase in the ratio of gastroesophageal reflux disease (GERD).^1,2^ In European countries, this transition occurred in prior decades and was related to increased erosive GERD and Barrett’s esophagus.^3,4^ While an increase in the prevalence of GERD in Japanese adults has been described,^5^ the real prevalence of Barrett’s esophagus in Japan is unclear because the diagnostic criteria for Barrett’s esophagus differ from country to country. In Japan, Barrett’s esophagus is diagnosed endoscopically.^5^ The Montreal definition also includes endoscopically suspected esophageal metaplasia without the requirement of biopsy.^6^

On the other hand, Barrett’s esophagus, which is diagnosed using histology, is the only recognized precursor lesion of esophageal adenocarcinoma in western countries.^3,4^ The increase of esophageal adenocarcinoma is coincident with the decline in *H. pylori* in the USA and is remarkable.^7^ Barrett’s cancer is also now increasing in Japan.^8^

The Z line is defined as the squamocolumnar junction where the squamous mucosa of the esophagus and columnar mucosa of the stomach meet. An irregular Z line represents the presence of columnar tongues >1 cm in length extending proximal to the gastroesophageal junction and has been reported at a prevalence of 10%-15% in those undergoing esophagogastroduodenoscopy (EGD) in western countries.^9^ The prevalence of intestinal metaplasia in those with an irregular Z line has been reported to be as high as 44%.^10,11^ The increase in GERD in the Japanese population has been associated with increased endoscopic Barrett’s esophagus, including an abnormal Z line.^12-14^

The health of the gastric mucosa has progressively changed in Japan. This is reflected in the documented increase in the gastric acid secretion levels in healthy young Japanese adults between 1995 and 2014^15^ and even in patients with severe gastric atrophy due to advanced age.^16^ This study examined changes in the endoscopic appearance of the esophageal–gastric junction and gastric mucosa in young Japanese adults in the last 15 years.

## Materials and Methods

### Study Design

This was a retrospective study based on endoscopic examinations performed in a single endoscopy center (Showa Inan General Hospital, Japan). Showa Inan General Hospital’s ethics committee approved the retrospective study protocol (no. 2021-08) on January 15, 2021. All patients had given written informed consent for the original procedures. The study was conducted in accordance with the latest version of the Declaration of Helsinki. Individuals cannot be identified from the data presented.

### Patients

Patients between 19 and 30 years in whom diagnostic EGD was performed for upper gastrointestinal symptoms and anemia in Showa Inan General Hospital between January 2006 and December 2020 were retrospectively examined. The indications for EGD were upper gastrointestinal symptoms, gastric cancer screening and anemia. Patients’ clinical features and EGD findings were obtained from electronic charts.

### Procedures

Esophagogastroduodenoscopy was performed using standard upper endoscopes (Olympus, Tokyo, Japan) by experienced gastroenterologists.

Propofol was nurse administered by bolus injection at an initial dose of 120 mg. About 20 or 40 mg of propofol was added to achieve an adequate sedation level, defined as when the subject had passed through the following sequence: eyes closing, 1 or 2 yawns, and cessation of body movements. All patients had cardiovascular monitoring and continuous oximeter measurements. Monitoring and adverse events such as respiratory suppression, hypotension, agitation, and arrhythmias were recorded by a registered nurse. When oxygen desaturation (SpO_2_ <90%) lasted for more than 20 seconds, supplemental oxygen was given.

### Definitions

**Figure 1.**
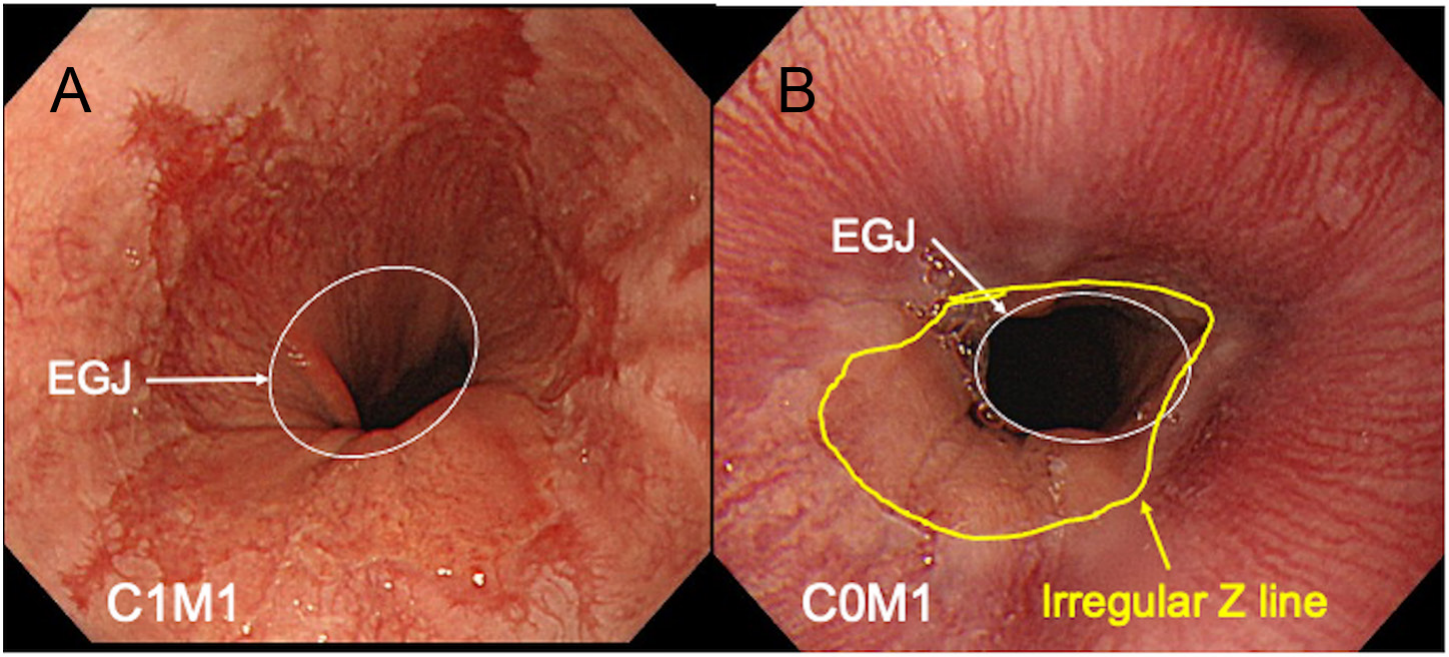
(A) A representative image of endoscopic Barrett’s esophagus (C1M1) in the Prague classification is shown. The white circle shows the esophagogastric junction (EGJ). (B) A representative image of endoscopic Barrett’s esophagus (C0M1) in the Prague classification is shown. The white circle shows the EGJ. The yellow line shows an irregular Z line.

The demarcation line between the squamous mucosa and columnar gastric mucosa represents the squamocolumnar junction, and it is defined as the “Z line.” The palisade vessels in the lower esophagus penetrate deeply inside the gastric wall and are no longer endoscopically visible. The site of this disappearance was defined as the esophagogastric junction.^5^ The distance from the Z line (squamocolumnar junction) to the esophagogastric junction was defined as endoscopic Barrett’s esophagus in Japan.^5^ The extent of endoscopic Barrett’s esophagus was assessed using “Prague C & M criteria” (Figure 1A and 1B). The extent of metaplasia above the esophagogastric junction was described using the circumferential (C) and maximal tongue (M) in centimeters.^17^ In addition, the extent of reflux esophagitis was graded using the Los Angeles classification.^18^

Fundic gland polyps were defined as endoscopically proven polyps of the gastric body and fundus that were 1-5 mm, sessile, and domed shaped with the color of normal mucosa.

### Data Collection and Evaluation

Endoscopic findings including the degree of reflux esophagitis and endoscopic Barrett’s esophagus were classified by Atsushi Morita who was blinded to the categorized groups. The ratio and degree of reflux esophagitis and endoscopic Barrett’s esophagus as well as other endoscopic findings were compared between 2006-2012 and 2013-2019. In addition, the characteristics of gastric fundic gland polyps were examined histopathologically, and serum gastrin levels were measured in a subset of patients with gastric fundic gland polyps.

### Histopathology

Biopsy specimens of gastric fundic gland polyps were obtained and stained with hematoxylin and eosin and Giemsa’s staining solution for histologic examination.

### Statistical Analyses

Statistical differences were analyzed by chi-square tests of independence and the Fisher’s exact test or the Student’s *t*-test. The Pearson coefficient of correlation analysis was performed to specifically examine the correlation between endoscopic Barrett’s esophagus and each type of endoscopic finding. *P*-values less than .05 were considered significant. Statistical analysis was performed by using GraphPad Prism Version 8.4.1 (GraphPad Software Inc., Tokyo, Japan).

## Results

### Baseline Characteristics of the Enrolled Patients

A total of 1845 patients were assessed, including 848 between 2006 and 2012 [400 males, mean age 26.5 (range 19-30) years] and 977 between 2013 and 2020 [433 males; mean age 26.2 (range 19-30) years]. The majority of patients enrolled underwent EGD for upper gastrointestinal symptoms. Patient demographics and indications were not significantly different between the 2 groups (Table 1). The use of proton pump inhibitors (PPIs) was rare. The percentages of patients with both reflux esophagitis and endoscopic Barrett’s esophagus significantly increased from the early period (2006-2012) to the late period (2013-2020) (19.5% vs. 29.5%, *P* < .001; 12.5% vs. 22.4%, *P* < .001). The presence of fundic gland polyps also significantly increased from the early to the late period (2006-2012, 3.4% vs. 2013-2020, 7.2%, *P* < .001). Other endoscopic findings such as esophageal hiatal hernia and chronic gastritis were similar between the 2 periods (Table 1). On the other hand, the prevalence of patients with gastric ulcer or duodenal ulcer was significantly decreased from 2006-2012 to 2013-2020 (2.0% vs. 0.8 %, *P* = .032; 4.8% vs. 2.5 %, *P* = .01).

### Endoscopic Barrett’s Esophagus

Endoscopic Barrett’s esophagus was identified in 262 patients: 82 patients [56 males; mean age 26.8 (19-30 years)] between 2006 and 2012 and 180 between 2013 and 2020 [107 males; mean age 26.4 (19-30 years)]. Patient demographics were not significantly different between the 2 groups (Table 2).

While the ratio of those with reflux esophagitis and endoscopic Barrett’s esophagus significantly increased from the early to the late periods, the rates of severity in Los Angeles classification or Prague classification were similar; the majority had a mild type, i.e., grade A (96.3% or 92%) or C0M1 (78% or 79.1%). The proportion of those with chronic gastritis, gastric ulcer, duodenal ulcer, fundic gland polyps, or esophageal hiatal hernia did not change significantly between the 2 periods (Table 2).

### Correlation Between Endoscopic Barrett’s Esophagus and Gastric Fundic Gland Polyps

**Figure 2.**
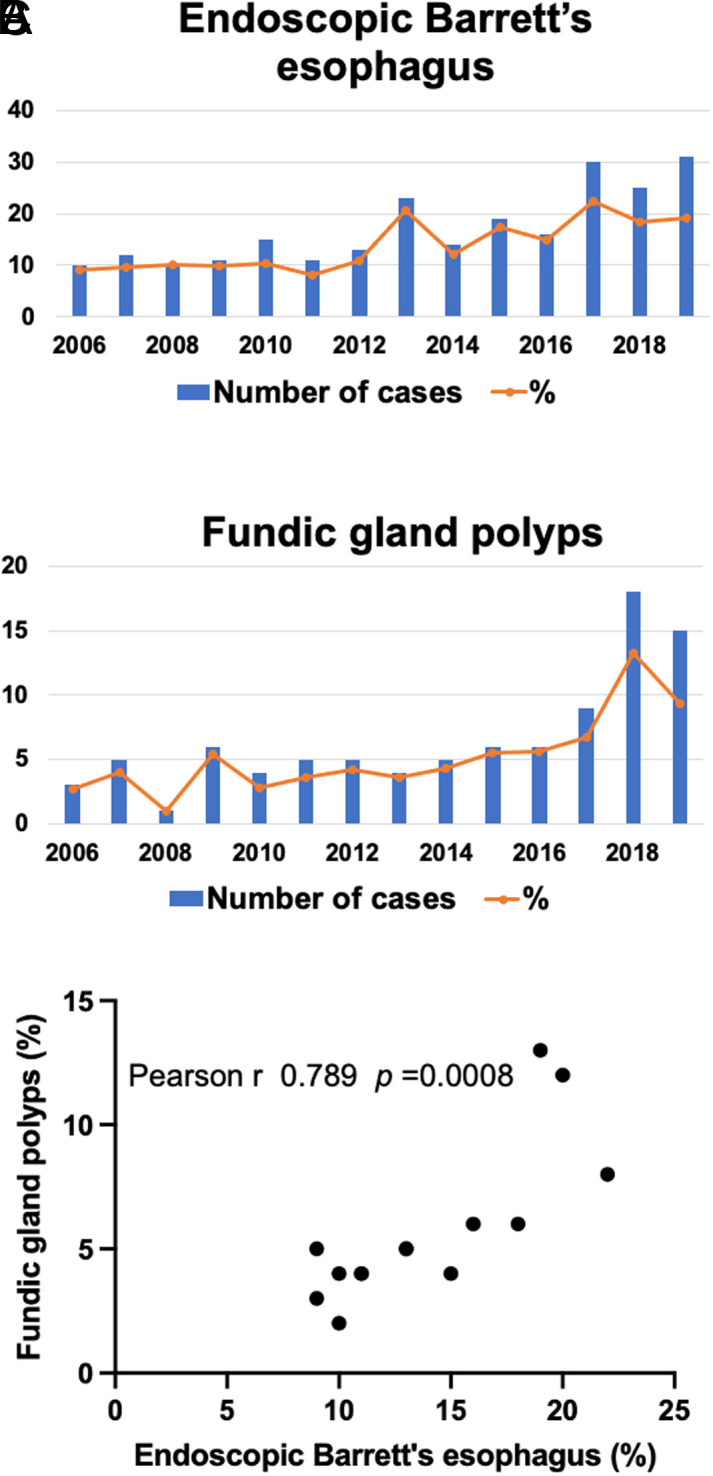
The yearly trends in endoscopic Barrett’s esophagus (A) and fundic gland polyps (B) for the past 15 years. (C) Pearson coefficient of correlation analysis revealed a significant correlation between these trends (*r* = 0.789, *P* = 0.0008).

Figure 2A and 2B shows yearly trends in both endoscopic Barrett’s esophagus and gastric fundic gland polyps in the last 15 years. The ratio of those with endoscopic Barrett’s esophagus and those with gastric fundic gland polyps increased and almost doubled from 2006 to 2019. Correlation analysis revealed that patients with endoscopic Barrett’s esophagus were significantly linked to those with gastric fundic gland polyps (*r* = 0.789, *P* = .0008) (Figure 2C).

***Pathohistological Analysis of Gastric Fundic Gland Polyps and Serum Gastrin Levels***

**Figure 3.**
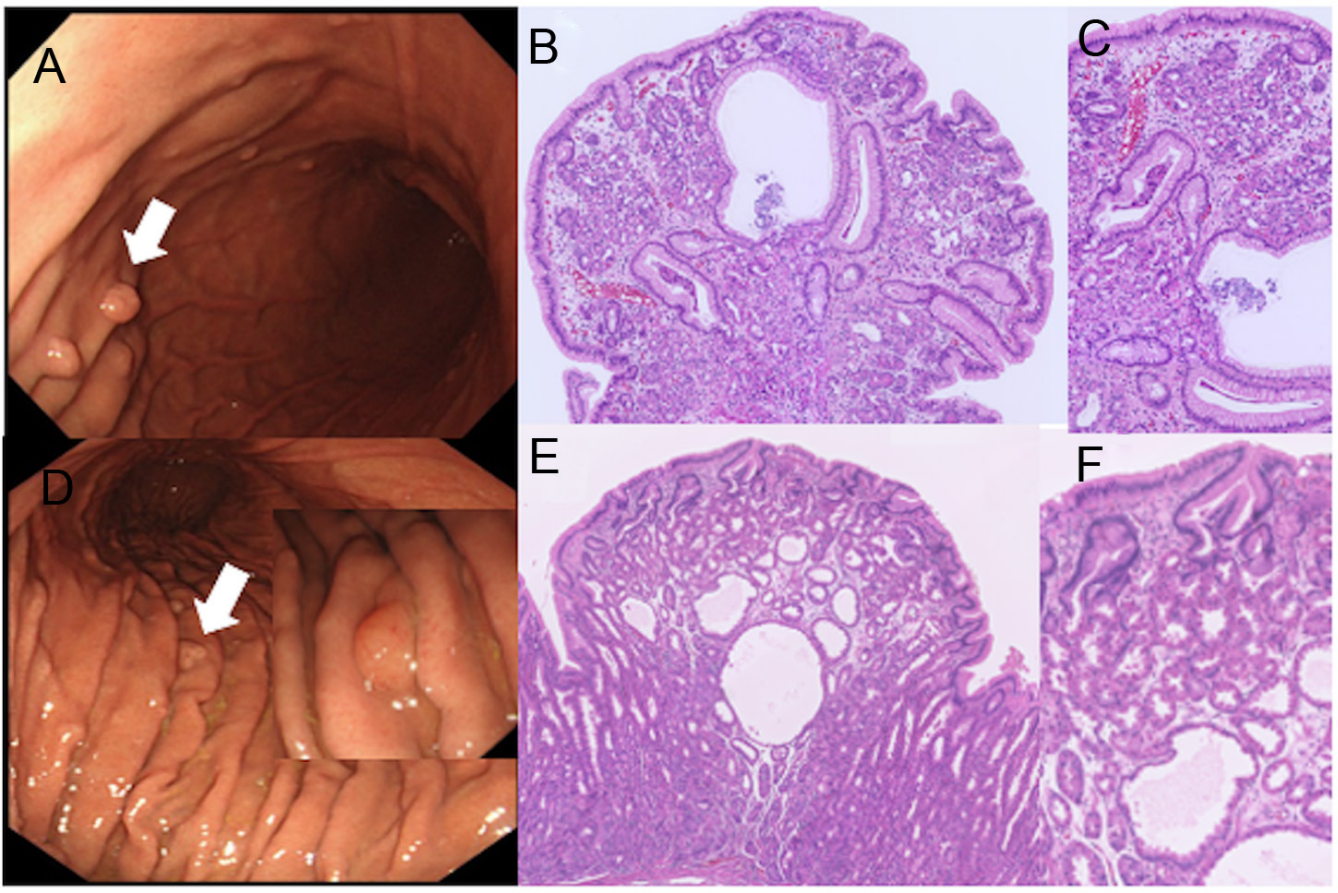
Endoscopic (A) and pathological findings of traditional fundic gland polyps (B, low resolution; C, high resolution). Endoscopic (D) and pathological findings of fundic gland polyp-like lesion (E, low resolution; F, high resolution).

Pathological examination was performed in 41 of 101 patients with gastric fundic gland polyps. The proportion of mucosal biopsy for the polyps between the 2 periods was similar (34% vs. 46%, *P* = .43) (Table 3). Histopathologically, traditional fundic gland polyps showed a mixture of fundic glands with distorted architecture, cysts lined with foveolar epithelium (foveolar cysts), and short or occasionally elongated foveolae (Figure 3A–C). On the other hand, fundic gland polyp-like lesions showed protrusions of parietal cells into the enlarged lumen of the fundic glands, revealing a serrated contour, fundic gland small cysts, and overlying enlarged foveolae without foveolar cysts (Figure 3D–F). Hypergastrinemia induced by PPI possibly may cause foveolar epithelial hyperplasia, which may be seen as white and flat elevated lesions. When multiple white and flat elevated lesions were observed, they were sometimes considered to be fundic gland polyps (Table 3).

The results of the pathological analysis showed that the prevalence of non-PPI-related traditional fundic gland polyps significantly increased from 40% (4/10) to 81% (25/31) between the 2 periods (*P* = .04) (Table 3). The serum gastrin levels in patients with traditional fundic gland polyps were normal (mean ± SD, 113 ± 36 pg/mL; range 75-200 pg/mL, n = 15) (the normal range <200 ng/mL), while those in patients with fundic gland polyp-like lesions were elevated (399 ± 118 pg/mL; range 229-500 pg/mL, n = 4).

## Discussion

Both reflux erosive esophagitis and endoscopic Barrett’s esophagus seen in young Japanese adults significantly increased in the last 15 years (Table 1). Interestingly, this study showed a significant correlation of trends in endoscopic Barrett’s esophagus and gastric fundic gland polyps despite a consistently rare use of PPIs (Figure 3). Pathological analysis in 41 of 101 patients with gastric fundic gland polyps confirmed that traditional fundic gland polyps in patients not taking PPIs significantly increased from 40% (4/10) to 81% (25/31) between the 2 periods (*P* = .04) (Table 3). Serum gastrin levels were normal in all patients with traditional fundic gland polyps. These findings suggest that overall gastric acid secretion may have increased in Japanese young adults over the past 15 years.

The prevalence of fundic gland polyps in Japanese patients undergoing endoscopy has increased from 4.4% in 1998 to 22.6% in 2010.^19^ In part, the increase is related to the decline in *H. pylori* infection, as fundic gland polyps are rarely, if ever, present in *H. pylori*-infected stomachs. It has been previously suggested that when gastric acid secretion is decreased by a PPI, compensatory hypergastrinemia occurs, and this effect of gastrin contributes to the increase and new development of fundic gland polyps.^20^ However, PPIs are not available as over-the-counter medicine in Japan, and the use of patients on long-term PPI therapy was rare in both periods. Actually, only 4 (13%) of the 31 patients with fundic gland polyp-like lesions were taking PPIs. The results of pathological examination confirmed that there were few PPI users among Japanese patients aged 19-30 years enrolled in this study.

This pathological analysis also showed that traditional fundic gland polyps in patients significantly increased from 40% (4/10) to 81% (25/31) between the 2 periods (*P* = .04), unrelated to the use of PPIs. The factors linked to the development of fundic gland polyps were not identified in this study. We suspect that this phenomenon may be associated with a trend in Japan toward higher protein diets, which enhances gastrin secretion via stimulated G cells and thereby increases acid secretion.^21-23^ Although the fasting levels of gastrin were normal in all patients with traditional fundic gland polyps, we did not assess meal-stimulated gastrin levels.

In the young subjects enrolled in this study, reflux esophagitis or endoscopic Barrett’s esophagus was limited in extent to only grade A/B or C0M1, C0M2, C1M1, and C1M2. In addition, in this study, there was no significant difference in the proportion of patients with esophageal hiatal hernia between the 2 periods, which is thought to be an important factor of esophageal acid exposure.^24,25^ Therefore, our results in this study were not affected by any differences in hiatal hernia.

This study has some limitations. First, this study was retrospective. It was difficult to eliminate the influence of the patients’ clinical features and endoscopic findings to the results as confounding factors. Although the proportion of patients in whom the esophagogastric junction was well visualized by endoscopy were similar, at 78% (658/848) in the early period and 82% (805/977) in the later period, it may be not possible to eliminate the observation bias by endoscopists. Finally, this study was conducted at a local hospital.

In conclusion, the prevalence of both endoscopic Barrett’s esophagus and gastric fundic gland polyps among young Japanese adults has significantly increased in the last 15 years. In addition, the trend in endoscopic Barrett’s esophagus was significantly correlated with that of gastric fundic gland polyps. This correlation may suggest increased gastric acid secretion due to the trend toward higher protein consumption by young Japanese adults.

## Figures and Tables

**Table 1.** Baseline Characteristics and Clinical Features of Young Patients Undergoing Esophagogastroduodenoscopy

Group	2006-2012 (n = 848)	2013-2020 (n = 997)	*P*
*Demographics*			
Average age [SD] (range), years	26.5 [3.4] (19-30)	26.2 [3.2] (19-30)	.57
Gender (male) (%)	400 (47.2%)	433 (43.4%)	.12
*Indications*			.20
Upper gastrointestinal symptoms	837	990	
Anemia	11	7	
Proton pump inhibitor user	3 (0.4%)	5 (0.5%)	.75
*Endoscopic findings*			
Reflux esophagitis^*^	128/658 (19.5%)	237/805 (29.5%)	<.001
Endoscopic Barrett’s esophagus^*^	82/658 (12.5%)	180/805 (22.4%)	<.001
Esophageal hiatal hernia	50 (5.9%)	75 (7.5%)	.19
Chronic gastritis	100 (11.8%)	110 (11%)	.41
Gastric ulcer	17 (2.0%)	8 (0.8%)	.032
Duodenal ulcer	41 (4.8%)	25 (2.5%)	.01
Gastric fundic gland polyps	29 (3.4%)	72 (7.2%)	<.001

^*^Patients in whom the esophagogastric junction was well visualized.

**Table 2.** Baseline Characteristics and Their Clinical Features of Patients with Endoscopic Barrett’s Esophagus

Group	2006-2012 (n = 82)	2013-2020 (n = 180)	*P*
*Demographics*			
Average age [SD] (range), years	26.8 [3.0] (19-30)	26.4 [3.1] (19-30)	.68
Gender (male)	56 (68.3%)	107 (59.5%)	.18
*Endoscopic findings and their classification*			
Reflux esophagitis			.49
Grade A	79 (96.3%)	166 (92%)	
Grade B	3 (3.7%)	14 (8%)	
Grade C/D	0 (0%)	0 (0%)	
*Endoscopic Barrett’s esophagus*			.52
C0M1	64 (78.0%)	142 (79.1%)	
C0M2	2 (2.4%)	8 (4.4%)	
C1M1	13 (15.9%)	25 (13.9%)	
C1M2	3 (3.7%)	5 (2.5%)	
Esophageal hiatal hernia	17 (20.7%)	39 (22.1%)	.80
Chronic gastritis	11 (13.4%)	16 (8.9%)	.27
Gastric ulcer	4 (4.9%)	2 (1.3%)	.089
Duodenal ulcer	4 (4.9%)	6 (3.2%)	.51
Gastric fundic gland polyps	6 (7.3%)	13 (7.0%)	.92

The severity of reflux esophagitis (Grade A, B, C, and D) was graded according to the Los Angeles classification.^18^ The extent of endoscopic Barrett’s esophagus was graded according to “Prague C & M criteria” assessing the circumferential (C) and maximum (M) extent of metaplasia above the esophagogastric junction in centimeters.^17^

**Table 3.** Pathological Findings in 41 of 101 Patients with Gastric Fundic Gland Polyps

Pathological Findings	2006-2012 (n = 10)	2013-2020 (n = 31)	*P*
Proportion of biopsy	34% (10/29)	46 % (31/72)	.43
Traditional fundic gland polyps	40% (4/10)	81% (25/31)	.04
Fundic gland polyp-like lesion	20% (2/10)	9.5% (3/31)	.58
Foveolar hyperplasia	40% (4/10)	9.5% (3/31)	.048
